# Automatic information extraction from childhood cancer pathology reports

**DOI:** 10.1093/jamiaopen/ooac049

**Published:** 2022-06-16

**Authors:** Hong-Jun Yoon, Alina Peluso, Eric B Durbin, Xiao-Cheng Wu, Antoinette Stroup, Jennifer Doherty, Stephen Schwartz, Charles Wiggins, Linda Coyle, Lynne Penberthy

**Affiliations:** Oak Ridge National Laboratory, Oak Ridge, Tennessee, USA; Oak Ridge National Laboratory, Oak Ridge, Tennessee, USA; College of Medicine, University of Kentucky, Lexington, Kentucky, USA; School of Public Health, Louisiana State University Health Sciences Center, New Orleans, Louisiana, USA; Rutgers Cancer Institute of New Jersey, New Brunswick, New Jersey, USA; Huntsman Cancer Institute, University of Utah, Salt Lake City, Utah, USA; Fred Hutchinson Cancer Research Center, Epidemiology Program, Seattle, Washington, USA; University of New Mexico, Albuquerque, New Mexico, USA; Information Management Services Inc., Calverton, Maryland, USA; National Cancer Institute, Bethesda, Maryland, USA

**Keywords:** pediatric cancer, cancer pathology reports, information extraction, machine learning

## Abstract

**Objectives:**

The International Classification of Childhood Cancer (ICCC) facilitates the effective classification of a heterogeneous group of cancers in the important pediatric population. However, there has been no development of machine learning models for the ICCC classification. We developed deep learning-based information extraction models from cancer pathology reports based on the ICD-O-3 coding standard. In this article, we describe extending the models to perform ICCC classification.

**Materials and Methods:**

We developed 2 models, ICD-O-3 classification and ICCC recoding (Model 1) and direct ICCC classification (Model 2), and 4 scenarios subject to the training sample size. We evaluated these models with a corpus consisting of 29 206 reports with age at diagnosis between 0 and 19 from 6 state cancer registries.

**Results:**

Our findings suggest that the direct ICCC classification (Model 2) is substantially better than reusing the ICD-O-3 classification model (Model 1). Applying the uncertainty quantification mechanism to assess the confidence of the algorithm in assigning a code demonstrated that the model achieved a micro-F1 score of 0.987 while abstaining (not sufficiently confident to assign a code) on only 14.8% of ambiguous pathology reports.

**Conclusions:**

Our experimental results suggest that the machine learning-based automatic information extraction from childhood cancer pathology reports in the ICCC is a reliable means of supplementing human annotators at state cancer registries by reading and abstracting the majority of the childhood cancer pathology reports accurately and reliably.

## INTRODUCTION

Cancer is the leading cause of death by disease in American children ages 0–19 years.^1^ Each year, nearly 16 000 children in the United States and over 300 000 children globally are diagnosed with cancer.^2^^,^^3^ Analysis of the population-level data for childhood cancers will increase the understanding of the factors that cause cancer and shed light on factors that may help protect against cancer, thus providing evidence to guide public health recommendations and identify and develop improved treatments. Further rapid identification and classification of cases might be used to enhance enrollment for clinical trials for ultra-rare pediatric cancers, thereby enabling access to state-of-the-art care to a wider set of childhood cancer patients.

Cancer pathology reports are an excellent resource for such studies. A pathology report is a medical document written by a pathologist that contains the diagnosis determined by examining cells and tissues under a microscope. The reports include information about the topography (site of origin) and the morphology (histology and behavior) of the tumor. Automatic information extraction is the machine learning (ML)-based method that abstracts the findings using the standardized codes.

We have been researching automatic information extraction and abstraction from cancer pathology reports based on the International Classification of Diseases for Oncology, third edition (ICD-O-3)^4–7^ coding standard. Recent advances in AI have enabled us to establish robust natural language processing and text comprehension algorithms, which could help mitigate the overhead of manually curating data. We have demonstrated that the deep learning (DL) models exhibited state-of-the-art performance compared against traditional ML-based and rule-based approaches. However, to the best of our knowledge, the efficacy of using the existing automatic information extraction models for childhood cancer pathology reports has not been studied.

The difficulties of applying the existing automatic information extraction model to the cancer pathology reports from childhood cancers originated from the differences of prevalent cancer types between adult and pediatric cancer cases. The most prevalent adult cancers are breast, lung and bronchus, prostate, and colorectal cancers.^8^ In contrast, the most common childhood cancers are leukemia, lymphoma, and tumors of the central nervous system.^9^ Cancers more prevalent in adults are underrepresented among the children, and—likewise—the cancers that are more prevalent in children are underrepresented among the adults. Because the number of adult cancers is substantially higher than the number of childhood cancers, the ML model trained using the entire cancer pathology data corpus is likely biased toward adult cancers. Consequently, this discrepancy may manifest downstream as a classification performance decrease for the childhood cancer model.

Also, studies^3^^,^^9^^,^^10^ suggest that the classification of childhood cancers should be based on morphology rather than topography. The ICD-O-3 is designed to categorize primarily by the site of origin, which is suitable for representing adult cancers. The International Classification of Childhood Cancer (ICCC),^9^ developed under the auspices of the International Agency of Research of Cancer (IARC), the International Association of Cancer Registries, and the Société Internationale d’Oncologie Pédiatrique (SIOP), is designed to emphasize the histology of tumors and leverages a combination of site and histology to characterize and classify childhood cancers. The information extraction from childhood cancer pathology reports should emphasize the morphology rather than the primary site of origin for these cancers.

The present study aims to develop an optimal ML model for automatic information extraction for pediatric cancer pathology reports based on ICCC coding and to establish a high-precision model by applying the uncertainty quantification (UQ) mechanism, which is critical for state cancer registries.

To that end, this article (1) developed a model of automatic information extraction from childhood cancer pathology reports based on ICCC, which—to the best of our knowledge—is the first AI/ML model for pediatric cancers; (2) presents results of a model trained on a large volume (29 206 cases) of pediatric cancer cases from 6 state cancer registries; (3) optimized the model for classifying childhood cancer pathology reports; and (4) describes the model calibration using UQ to support human annotators with high precision.

## MATERIALS AND METHODS

### Data sources

This study’s data set consisted of unstructured text in pathology reports from 6 state cancer registries: the Kentucky Cancer Registry (KCR), Louisiana Tumor Registry (LTR), New Jersey State Cancer Registry (NJSCR), New Mexico Tumor Registry (NMTR), Seattle Cancer Registry (SCR), and the Utah Cancer Registry (UCR). KCR, LTR, NMTR, SCR, and UCR participate in the National Cancer Institute’s (NCI’s) Surveillance, Epidemiology, and End Results (SEER) program. The study was executed according to the institutional review board protocol DOE000619, approved by the US Department of Energy (DOE) Institutional Review Board on April 6, 2021 (initial approval on September 23, 2016). From the data of millions of e-path reports from the cancer registries, we selected cases in which a cancer patient was diagnosed before they were 20 years old.

The gold standard of the abstraction of information extracted from e-path reports is the Cancer/Tumor/Case (CTC) database, which stores all diagnostic, staging, and treatment information for reportable cancers in the SEER Data Management System. Notably, the CTC provides the abstraction of the e-path reports in terms of the ICD-O-3, such as primary cancer site, laterality, histology, and behavior, but does not contain ICCC coding of the cases. Instead, the ICCC codes are being recoded from the ICD-O-3.

### ICCC recoding

The NCI SEER provides tables that list ICCC codes and their corresponding ICD-O-3 site and histology codes.^11^ Notably, a few slight modifications were made to the coding standards to allow for new and expanded coding of cancers. The latest ICCC recode standard is the “ICCC, Third Edition, based on ICD-O-3/IARC 2017,” which we used in our studies. We chose the ICCC regular recoding as our truth labeling and inference protocol, which consists of 12 main groups and 46 subgroups. Table 1 lists the codes and descriptions for the 12 main groups and 46 subgroups.

**Table 1.** ICCC (a) main and (b) subgroup codes and definitions based on the ICCC third edition

(a)	
Code	Description
01	Leukemias, myeloproliferative, and myelodysplastic diseases
02	Lymphomas and reticuloendothelial neoplasms
03	CNS and miscellaneous intracranial and intraspinal neoplasms
04	Neuroblastoma and other peripheral nervous cell tumors
05	Retinoblastoma
06	Renal tumors
07	Hepatic tumors
08	Malignant bone tumors
09	Soft tissue and other extraosseous sarcomas
10	Germ cell tumors, trophoblastic tumors, and neoplasms of gonads
11	Other malignant epithelial neoplasms and malignant melanomas
12	Other and unspecified malignant neoplasms
999	Not classified by SEER or in situ

(b)			
Code	Description	Code	Description
011	Lymphoid leukemias	081	Osteosarcomas
012	Acute myeloid leukemias	082	Chondrosarcomas
013	Chronic myeloproliferative diseases	083	Ewing tumor and related sarcomas of bone
014	Myelodysplastic syndrome and other myeloproliferative diseases	084	Other specified malignant bone tumors
015	Unspecified and other specified leukemias	085	Unspecified malignant bone tumors
021	Hodgkin lymphomas	091	Rhabdomyosarcomas
022	Non-Hodgkin lymphomas	092	Fibrosarcomas, peripheral nerve sheath tumors, and other fibrous neoplasms
023	Burkitt lymphoma	093	Kaposi sarcoma
024	Miscellaneous lymphoreticular neoplasms	094	Other specified soft tissue sarcomas
025	Unspecified lymphomas	095	Unspecified soft tissue sarcomas
031	Ependymomas and choroid plexus tumor	101	Intracranial and intraspinal germ cell tumors
032	Astrocytomas	102	Malignant extracranial and extragonadal germ cell tumors
033	Intracranial and intraspinal embryonal tumors	103	Malignant gonadal germ cell tumors
034	Other gliomas	104	Gonadal carcinomas
035	Other specified intracranial and intraspinal neoplasms	105	Other and unspecified malignant gonadal tumors
036	Unspecified intracranial and intraspinal neoplasms	111	Adrenocortical carcinomas
041	Neuroblastoma and ganglioneuroblastoma	112	Thyroid carcinomas
042	Other peripheral nervous cell tumors	113	Nasopharyngeal carcinomas
050	Retinoblastoma	114	Malignant melanomas
061	Nephroblastoma and other nonepithelial renal tumors	115	Skin carcinomas
062	Renal carcinomas	116	Other and unspecified carcinomas
063	Unspecified malignant renal tumors	121	Other specified malignant tumors
071	Hepatoblastoma and mesenchymal tumors of the liver	122	Other unspecified malignant tumors
072	Hepatic carcinomas	999	Not classified by SEER or in situ
073	Unspecified malignant hepatic tumors		

### Childhood cancer pathology report data corpus

**Figure 1.**
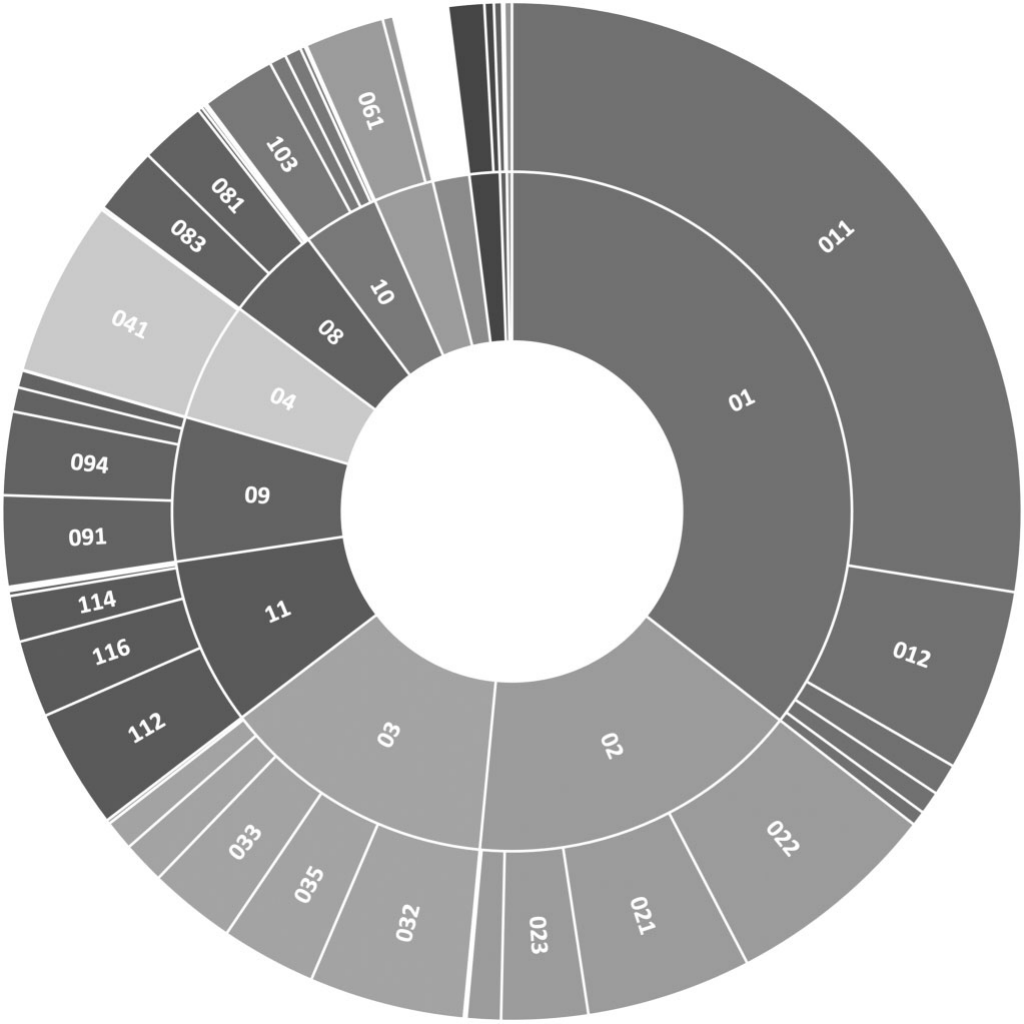
Number of childhood cancer pathology reports by ICCC main and subgroup codes.

The total number of childhood cancer pathology reports in our data corpus is 29 206 from 11 274 patients. Figure 1 illustrates the number of cases per each ICCC code. Leukemias (01) and lymphomas (02) represent more than half of all childhood cancers. Leukemia is the most prevalent cancer in children, and this finding is consistent with existing research.^12^ Lymphoid leukemia (011) is the most prevalent leukemia and represents more than 25% of childhood cancer cases. Among lymphomas, non-Hodgkin lymphoma (022) is the most prevalent type. Note that the figure illustrates the severe class imbalance within this data set.

**Figure 2.**
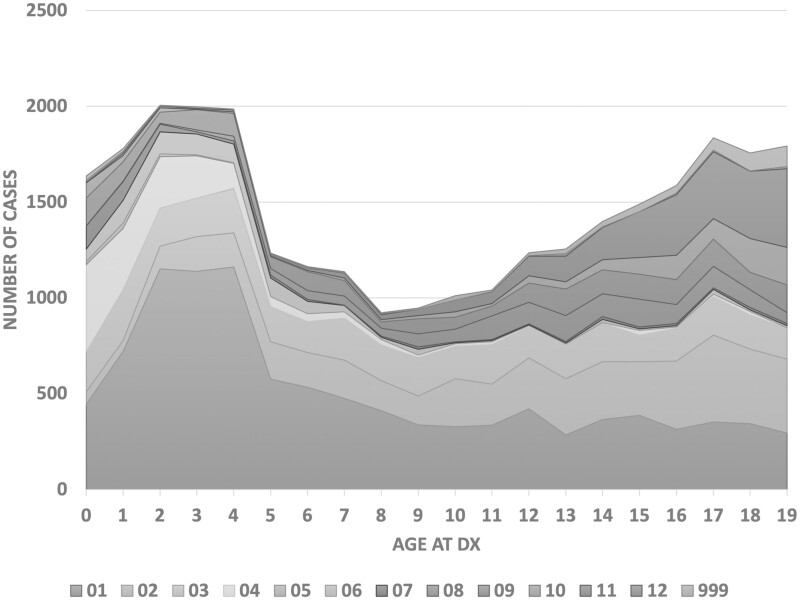
Number of childhood cancer pathology reports by ICCC main codes and age at diagnosis.

Figure 2 illustrates the number of cases per ICCC code sorted by a patient’s age at diagnosis. Leukemia (01) is more common in younger patients (ages 0–4) but diminishes among older children. Similar patterns occurred for patients with neuroblastoma (04). In contrast, the incidence of lymphomas (02) was highest among adolescents. Germ cell tumors (10) and other malignant epithelial tumors and melanomas (11) are also most common among young adolescents. Note that the number of cancer cases between ages 5 and 11 is considerably lower than for the other age groups. The observations and findings are consistent with the reports and statistics from other studies,^8^^,^^13^ which implies that the data set from the 6 population-based registries in SEER included in this study reflects the real-world situation.

### ML models for text classification

TextCNN^4^^,^^14^ is one of the most successful and widely used convolutional neural network (CNN) models for text comprehension and classification. It consists of 3 parts: word embedding, 1D convolution, and a fully connected decision layer. Word embedding is a learned representation of terms and words to map a set of words onto vectors of numerical representations with the same semantic meaning and similar observation. The 1D convolution layer has a series of 1D convolution filters that have latent representations to articulate the features in the word vectors of documents. The features found are passed to the fully connected layer to make inferences. MT-CNN^5^ extends TextCNN by adding a multitask learning (MTL) mechanism^15^ to the decision layer. A classifier learns multiple tasks simultaneously and finds an optimal latent representation to solve a series of related tasks. The MTL helps find more generalized solutions than single-task models, thus yielding higher task performance. We have successfully developed an MT-CNN model for automatic information extraction based on ICD-O-3 and verified that the CNN model has competitive task performance while exhibiting prompt training and inference time.^16^

### Automatic information extraction based on ICCC

The following subsections describe 2 models that we designed and tested for this study along with 2 scenarios for each model.

#### Model 1: ICD-O-3 classification then ICCC recoding approach

The first model involves the classification of ICD-O-3. Generally, the ICCC coding is a recoding based on the site, histology, and behavior from the ICD-O-3 codes. Therefore, the automatic information extraction from childhood cancer pathology reports can be accomplished using the existing classification model^5^ for cancer patients of all ages. This approach saves the time and effort required to develop a new model for classifying cancer pathology reports based exclusively on the ICCC coding.

However, some factors may cause a decrease in classification accuracy. First, ICCC includes 47 codes, whereas ICD-O-3 consists of more than 300 site codes and 600 histology codes.^17^ Designing and training an ML/DL model with that many labels could be overly complex and prone to error. Second, as stated earlier, certain cancer types are more prevalent in adults than in children and vice versa; moreover, cancer is more prevalent in adults, generally. Consequently, the model trained on the entire corpus of cancer patients could be skewed more toward the reports from adult patients.

We developed 2 scenarios to evaluate if we can achieve better accuracy by limiting the scope to pathology reports of childhood cancers.

1. Model 1(a): ICD-O-3 classification model is trained by cancer pathology reports from all age groups and then recoded for ICCC
2. Model 1(b): ICD-O-3 classification model is trained by the childhood cancer pathology reports only and then recoded for ICCC

#### Model 2: Direct ICCC classification approach

The second model involves classifying ICCC codes directly from the cancer pathology reports. The new model was trained on ICCC data and may have higher classification accuracy because it only deals with 47 classes, whereas the ICD-O-3-based models must contend with numerous class labels. This approach required us to train and deploy another ML model specifically for childhood cancer pathology reports, which required extra effort and resources.

In addition, we conducted a further study based on the consensus recommendation from the Childhood Cancer Data Initiative’s (CCDI’s) advisory group that the upper age limit of diagnosis be up to 39 years old for certain childhood/pediatric cancers. Table 2 lists the cancer types to be regarded as pediatric cancers at this upper age limit per CCDI’s suggestion. Note that 1055 cases fall into these categories, which is a relatively small number.

**Table 2.** List of cancers diagnosed between ages 20 and 39 that could be regarded as pediatric cancers per CCDI and the number of cases that are augmented to the training of Model 2(b)

Code	Description	No. of cases
061	Nephroblastoma and other nonepithelial renal tumors	26
071	Hepatoblastoma and mesenchymal tumors of the liver	11
081	Osteosarcoma	301
082	Chondrosarcoma	232
083	Ewing tumors and related sarcoma of the bone	193
084	Other specified malignant bone tumors	90
085	Unspecified malignant bone tumors	23
091	Rhabdomyosarcomas	179

We developed 2 scenarios to quantify the effect of augmenting the data per CCDI’s recommendation.

1. Model 2(a): ICCC classification model is trained by the childhood cancer pathology reports
2. Model 2(b): ICCC classification model is trained by the childhood cancer pathology reports with augmentation suggested by CCDI

**Figure 3.**
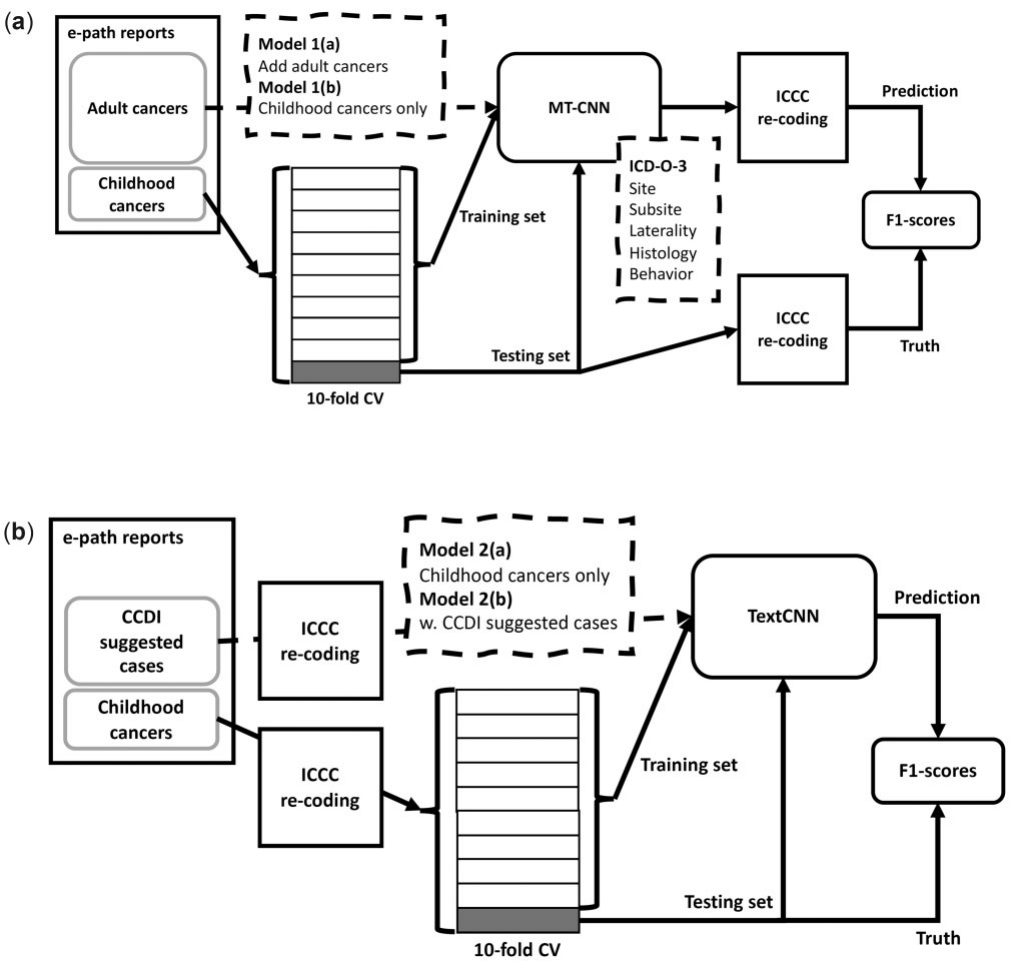
Model architecture for ICCC classification from childhood cancer pathology reports. (A) Model 1: ICD-O-3 classification then ICCC recoding. (B) Model 2: direct ICCC classification.

Figure 3 illustrates the architectures of the 2 models (and 4 scenarios) that we designed and evaluated.

### Uncertainty quantification

The purpose of automatic information extraction is to either assist human observers with a second opinion or automate coding where feasible to enable humans to focus on cases that are more complex or challenging. To this end, the most important feature that the model should possess to achieve the objective is a highly reliable and accurate decision from the model. Inaccurate second opinions (from the model) may distract human observers and even degrade the process’ performance. If the model’s decision is incorrect, then review is needed, which limits the efficiency and benefit of using an automated process. UQ, which is vital to the process, assigns a confidence estimate to the machine-assigned code to allow a human to determine whether additional review is necessary, thus minimizing human labor.

In this article, we propose a post-training threshold approach based on the estimation of a confidence score from the softmax-predicted probabilities in the validation set (rather than the training set).

Let $Y=(y_{1},\ldots ,y_{n})$ be the softmax-predicted probabilities for the n classification labels. The confidence score is estimated as the conditional distribution of a correct classification via the Bayes theorem for a binary variable (in which being correct and incorrect are mutually exclusive outcomes):

$$p\left(\mathrm{correct}|y\right)=\frac{p\left(y|\mathrm{correct}\right)\cdot p\left(\mathrm{correct}\right)}{p\left(y|\mathrm{correct}\right)\cdot p\left(\mathrm{correct}\right)+p\left(y|\mathrm{incorrect}\right)\cdot p\left(\mathrm{incorrect}\right)}$$

The marginal probabilities p(correct) and p(incorrect) are called priors and are estimated as the corresponding relative frequencies (ie, the total number of correct or incorrect decisions divided by the total number of cases).

The conditional probabilities p(y|correct) and p(y|incorrect) are estimated from the data by modeling the conditional quantile functions $F_{y|\mathrm{correct}}^{-1}(\tau |\mathrm{correct})$ and $1-F_{y|\mathrm{incorrect}}^{-1}\left(\tau |\mathrm{incorrect}\right)$ for a selected percentile point, τ.

### Experimental setup

We designed a comparison study to determine the classification accuracies of the models described above. F1 scores, a widely accepted metric for information retrieval, are used as the performance benchmark. Because of the severe class imbalance of the data corpus, we employed both micro-averaged and macro-averaged F1 scores. The micro-F1 is weighted equally to the individual cases, whereas the macro-F1 is weighted equally to the class label. If the ML model was favorable to the prevalent class labels but did not work well with the samples from minor classes, then the macro-F1 score would be lower than the micro-F1 score.

To compensate for the limited availability of childhood cancer pathology reports in the data corpus, we chose to perform 10-fold cross-validation tests. We utilized the StratifiedKFold function available in the scikit-learn library.^18^ The TextCNN and MT-CNN model training used the Keras/TensorFlow platform.^19^^,^^20^

## RESULTS

We performed 2 experiments: the first experiment aimed to develop an optimal classification model for information extraction based on ICCC, and the second experiment aimed to establish a reliable model by adopting the UQ mechanism.

### Developing an optimal classification model

Table 3 lists the associated task performance for classifying ICCC codes from the childhood cancer pathology reports for both micro-averaged and macro-averaged F1 scores. Model 2 recorded substantially higher scores than Model 1, which implies that the models trained by the ICCC codes performed better than the models for classifying ICD-O-3 codes and recoding them to ICCC. Macroscores (0.701–0.843) showed more improvement than microscores (0.882–0.936), which indicates that Model 2 performed better for the underrepresented class labels. Model 1(b), trained only on the childhood cancer cases, performed slightly better than Model 1(a), which incorporated adult cancer cases. However, the difference was negligible. Performance differences between Models 2(a) and 2(b) were also negligible.

**Table 3.** Classification accuracy scores per each ICCC code in F1 metric

Code	1(a)	1(b)	2(a)	2(b)	# cases	UQ	# UQ
011	0.94	0.95	0.97	0.97	8042	0.99	7171
012	0.88	0.91	0.93	0.93	1694	0.98	1450
013	0.90	0.89	0.94	0.93	294	0.98	248
014	0.62	0.57	0.71	0.66	137	0.92	61
015	0.33	0.49	0.76	0.75	212	0.92	127
021	0.94	0.94	0.96	0.96	1530	0.99	1380
022	0.75	0.80	0.87	0.88	1993	0.96	1498
023	0.84	0.86	0.90	0.90	801	0.98	641
024	0.96	0.96	0.98	0.97	316	1.00	299
025	0.07	0.00	0.15	0.17	25	0.25	14
031	0.89	0.89	0.93	0.93	391	0.99	333
032	0.88	0.90	0.92	0.93	1441	0.99	1238
033	0.90	0.90	0.93	0.92	803	0.99	681
034	0.52	0.58	0.76	0.76	278	0.93	156
035	0.82	0.85	0.91	0.92	881	0.98	704
036	0.00	0.24	0.60	0.61	36	0.90	16
041	0.96	0.97	0.98	0.98	1639	1.00	1558
042	0.64	0.55	0.73	0.74	26	1.00	12
050	0.92	0.95	0.99	0.99	71	1.00	68
061	0.97	0.97	0.98	0.98	736	1.00	694
062	0.87	0.89	0.94	0.95	99	0.99	87
071	0.96	0.96	0.98	0.97	334	0.99	315
072	0.89	0.87	0.92	0.92	87	0.98	73
081	0.96	0.97	0.98	0.98	617	1.00	581
082	0.80	0.53	0.81	0.80	40	0.93	35
083	0.79	0.83	0.87	0.89	627	0.97	433
084	0.73	0.45	0.76	0.85	30	0.98	20
085	0.16	0.23	0.38	0.60	25	0.82	10
091	0.93	0.93	0.96	0.95	840	0.99	764
092	0.71	0.74	0.83	0.82	159	0.97	102
093	0.83	0.00	0.29	0.50	6	0.00	1
094	0.72	0.73	0.83	0.84	772	0.97	508
095	0.57	0.58	0.77	0.78	225	0.96	128
101	0.70	0.72	0.84	0.85	155	0.99	105
102	0.77	0.83	0.86	0.87	152	0.98	101
103	0.94	0.94	0.96	0.96	688	1.00	619
104	0.56	0.65	0.80	0.84	44	0.96	24
105	0.44	0.36	0.80	0.73	18	1.00	6
111	0.64	0.73	0.80	0.76	16	0.77	8
112	0.99	0.99	0.99	0.99	1112	1.00	1096
113	0.92	0.89	0.95	0.93	46	0.99	40
114	0.95	0.89	0.98	0.97	427	0.99	392
115	0.59	0.59	0.86	0.77	23	1.00	9
116	0.88	0.92	0.96	0.96	715	1.00	639
121	0.59	0.58	0.88	0.82	75	0.99	44
122	0.00	0.09	0.58	0.54	20	1.00	3
999	0.75	0.72	0.90	0.89	508	0.97	400
Micro-F1	0.882	0.896	0.935	0.936	29 206	0.987	24 892
Macro-F1	0.701	0.719	0.837	0.843	29 206	0.935	24 892

*Note*: Column 1(a) is the scores from Model 1(a), 1(b) is from Model 1(b), 2(a) is from Model 2(a), and 2(b) is from Model 2(b), “# cases” is the number of classified cases in the data corpus, UQ is the scores from Model 2(b) but without abstained cases based on the softmax UQ, and “# UQ” is the number of classified cases by the UQ model. Micro-averaged and macro-averaged F1 scores are at the bottom of the table.

The classification accuracy for each ICCC code was analyzed further, and the results are listed in Table 3. Overall, Model 2 recorded higher scores across the ICCC codes. The difference was higher for the underrepresented ICCC codes (eg, 015: Model 1[a] 0.33, Model 2[b] 0.75) than for the more prevalent codes/types (eg, 001: Model 1[a] 0.94, Model 2[b] 0.97). Also, the models performed better for the prevalent ICCC codes (eg, 021 [1530 cases]: Model 2[b] 0.96) than for the minor ones (eg, 025 [25 cases]: Model 2[b] 0.17). However, there were no significant differences between Models 1(a) and 1(b) or between Models 2(a) and 2(b).

### Establishing a reliable model

The UQ was applied to Model 2(b), which recorded the highest accuracy score. The abstention classifier was tuned to abstain on cases with confidence scores that were associated with softmax-predicted probabilities lower than 0.9. With the UQ mechanism, Model 2(b) achieved a micro-F1 score of 0.987 and a macro-F1 score of 0.935, and the model discarded only 14.8% of cases from the data corpus. Table 3 lists the accuracy scores for each ICCC code. With the softmax-based UQ, we achieved high accuracy scores across all ICCC labels while maintaining a low abstention rate. Still, there were a few ICCC code outliers in the table for which the model with UQ did not achieve high accuracy scores: unspecified lymphomas (025), unspecified malignant bone tumors (085), Kaposi sarcoma (093), and adrenocortical carcinomas (111).

## DISCUSSION

In terms of classification accuracy, the results in Table 3 support the argument that training the models for classifying ICCC codes showed improved accuracy and reliability over the method of using existing ICD-O-3 classification models and then performing ICCC recoding. Presumably, the lower performance on Model 1 is caused by the complexity of the ICD-O-3 classifications, which consist of more than 300 class labels associated with subsites and more than 600 class labels with histology. Moreover, in our data corpus, many of the ICD-O-3 class labels are severely underrepresented. In contrast, the ICCC consists of only 47 class labels. Note that even the ICCC distribution remains imbalanced because of the high prevalence of certain cancer types (leukemias and lymphomas) and the occurrence of “ultra-rare” pediatric tumors. However, the severity of this imbalance is substantially smaller than for the ICD-O-3 system as applied to pediatric cancers.

Performance degradation in information extraction models caused by underrepresented class labels in the data corpus is a critical issue in developing algorithms for automation in cancer surveillance. There is no definitive way to increase the sample size of cancer pathology reports from rare cancers given the rarity of pediatric tumors in general (16 000 cases in the United States per year). One might suggest special ML techniques, such as data synthesis, but it is well known that synthesizing free-form text data is not a trivial task.

One possible solution is to augment the training corpus with the reports from the subjects of age 20–39, as some ICCC sites are considered pediatric tumors even when occurring in adolescents or young adults. The results listed in Table 3 show the effects of adding those reports based on expert consensus in the CCDI community. The class labels that already had more than 100 samples in the corpus did not benefit from the augmentation because the model had already achieved high classification accuracy scores for those labels. However, substantial improvements were made for code 084—*Other specified malignant bone tumors* (from 0.76∼F1 to 0.85∼F1) and for code 085—*Unspecified malignant bone tumors* (from 0.38∼F1 to 0.60∼F1). Those 2 labels had a relatively small number of samples in the corpus: 30 cases for code 084 and 25 cases for code 085. Adding 90 cases for code 084 and 23 cases for code 085 increased the chance of learning features for correct decisions for those class labels.

Note, however, that Model 1(b) recorded higher accuracy scores than Model 1(a), which implies that simply adding adult cancer pathology reports did not improve accuracy. This makes sense because the corpus contained many more adult cancers than childhood cancers, and the prevalent adult cancers (eg, breast cancer) are rare in childhood. Thus, this simple addition may not improve the ML model’s performance for childhood cancer cases, which leads to the following question: can we improve the accuracy score if we curate the augmented data set by undersampling the breast and lung cancers or by downselecting the cancer types that are more prevalent in children? This would make an excellent future research topic. This solution of expanding the corpus based on codes, combined with expanding the training corpus by bringing in additional registries, might serve as a partial but nontrivial solution to reduce the class imbalance.

The application of the softmax-based UQ mechanism was successful. We demonstrated that Model 2(b) with the softmax-based UQ achieved 0.987—a nearly perfect micro-F1 score—while it abstained in only 14.8% of the cases. This result implies that the system could serve human annotators at state cancer registries. The model can process more than 85.2% of the childhood cancer pathology reports with confidence. Further manual review is needed for only 14.8% of the incoming data, which indicates that the model is highly reliable and potentially ready to use.

Several factors contribute to abstention of cases, such as the case difficulty, incompleteness of information on the data samples, or not enough information supplied to the classification model owing to a lack of training samples. One clue is that the abstention rate (ie, the number of abstained cases divided by the total number of cases in the class label) is considerably lower for the prevalent classes. For example, the abstention rate of code 011 was 0.108 (871/8042), whereas the abstention rate for code 014 was 0.555 (76/137). Increasing performance for those underrepresented classes is key to achieving a more reliable model.

Some questions remain. The choice of 0.9 as the threshold of probability for correct decision-making is entirely arbitrary and unlikely to be sufficiently robust to meet the high-quality standards of the surveillance community. The threshold value is subject to the tolerance level of wrong decisions by the state cancer registries and will determine the credibility of the data products from the registries. Follow-up statistical analyses of this threshold are required.

## CONCLUSION

In this article, we described our study of the classification of childhood cancer pathology reports in terms of the ICCC coding and established an automatic information extraction system for processing a massive volume of pathology reports suitable for state cancer registries. We tested the 2 models: (1) reusing the existing model for extracting ICD-O-3 codes and recoding them into ICCC and (2) developing a new model for extracting ICCC coding. We also experimented with a softmax-based UQ algorithm to evaluate model performance when discarding the minimum amount of ambiguous cases. Our findings suggest that the model for classifying ICCC coding with UQ is suitable for alleviating the workload of human annotators at state cancer registries.

## FUNDING

This work was supported in part by the Joint Design of Advanced Computing Solutions for Cancer (JDACS4C) program established by DOE and the NCI of the National Institutes of Health. This work was performed under the auspices of DOE by Argonne National Laboratory under Contract DE-AC02-06-CH11357, Lawrence Livermore National Laboratory under Contract DE-AC52-07NA27344, Los Alamos National Laboratory under Contract DE-AC5206NA25396, and Oak Ridge National Laboratory under Contract DE-AC05-00OR22725.

## AUTHOR CONTRIBUTIONS

H-JY carried out the problem conception, implementation, validation tests, and drafted manuscript. AP carried out the design of uncertainty quantification algorithm. EBD, X-CW, AS, JD, SS, CW, and LC carried out the data curation. EBD, X-CW, and LP helped to draft the manuscript. All authors read and approved the final manuscript.
